# A real-world pharmacovigilance analysis of ALK inhibitor-associated pleural and pericardial effusion using the FDA Adverse Events Reporting System (FAERS) database from 2013 to 2024

**DOI:** 10.1371/journal.pone.0330630

**Published:** 2025-08-19

**Authors:** Connor Frey

**Affiliations:** Department of Medicine, University of British Columbia, Vancouver, British Columbia, Canada; Institute of Medical Sciences, Banaras Hindu University, INDIA

## Abstract

**Introduction:**

The advent of anaplastic lymphoma kinase (ALK) inhibitors has revolutionized the treatment of ALK-rearranged malignancies, establishing these agents as vital components of precision oncology. Despite their proven efficacy in prolonging progression-free and overall survival, ALK inhibitors are associated with notable adverse events, particularly cardiopulmonary complications such as pleural and pericardial effusions.

**Methods:**

This study investigates the real-world prevalence and risk of these effusions associated with five ALK inhibitors, crizotinib, ceritinib, alectinib, brigatinib, and lorlatinib, through disproportionality analysis using the FAERS pharmacovigilance database.

**Results:**

The data revealed elevated reporting odds ratios (RORs) for pleural and pericardial effusions, with notable variability among the agents. Crizotinib exhibited RORs of 7.76 (95% CI: 6.60–9.12) and 9.00 (95% CI: 7.10–11.41) for pleural and pericardial effusions, respectively. Ceritinib demonstrated RORs of 7.36 (95% CI: 5.16–10.50) and 10.80 (95% CI: 6.79–17.19), respectively. Alectinib showed lower RORs of 4.76 (95% CI: 3.80–5.97) and 6.67 (95% CI: 4.92–9.04). Brigatinib displayed elevated RORs of 8.70 (95% CI: 6.58–11.52) and 7.87 (95% CI: 4.95–12.51). Lorlatinib presented the highest risk, with RORs of 8.61 (95% CI: 6.72–11.02) and 12.57 (95% CI: 9.08–17.38).

**Conclusions:**

This study highlights the critical need for vigilant pharmacovigilance and a multidisciplinary approach to balance the oncologic benefits of ALK inhibitors against their cardiopulmonary risks. By enhancing awareness and fostering proactive management, these findings aim to support the safe and effective use of ALK inhibitors in treating ALK-rearranged malignancies.

## Introduction

The development of anaplastic lymphoma kinase (ALK) inhibitors has significantly transformed the therapeutic landscape for ALK-rearranged malignancies, establishing these agents as a key therapeutic option in precision oncology [1–3]. ALK inhibitors function by targeting and inhibiting the ALK tyrosine kinase receptor, thereby disrupting aberrant signalling cascades that drive tumorigenesis [4–6]. In 2023, global sales were highest for alectinib and brigatinib at $1.65 billion each, followed by crizotinib and lorlatinib at $1.2 billion each, and ceritinib at $91 million. Despite their efficacy in controlling tumour growth and improving progression-free and overall survival, the clinical use of ALK inhibitors has been associated with a distinctive spectrum of adverse events, including cardiopulmonary complications [7–11]. Among these, the development of pericardial and pleural effusions warrants particular attention, as these conditions represent serious, albeit less frequently discussed, clinical challenges.

Pericardial effusion involves the pathological accumulation of fluid within the pericardial sac, while pleural effusion occurs when fluid build-up occurs within the pleural cavities. Both conditions may manifest across a spectrum of clinical severity, ranging from mild, subclinical presentations to life-threatening scenarios characterized by respiratory compromise and hemodynamic instability [12,13]. The pathophysiological mechanisms underpinning these effusions remain incompletely understood. Clinicians face diagnostic complexities in distinguishing these adverse events from disease progression or other etiologies, which is critical for timely and appropriate intervention.

This study comprehensively investigates the real-world prevalence of pericardial and pleural effusions associated with five widely used ALK inhibitors: crizotinib, ceritinib, alectinib, brigatinib, and lorlatinib. By aggregating and analyzing real-world evidence, this study aims to bridge existing knowledge gaps, providing oncologists, cardiologists, and pulmonologists with actionable insights for effectively managing these complications. Enhanced awareness and vigilance among healthcare providers can facilitate timely diagnosis and tailored therapeutic approaches, thereby preserving treatment continuity and improving the quality of life for patients. Ultimately, this research seeks to contribute to a more nuanced understanding of the safety profile of ALK inhibitors, balancing their substantial oncologic benefits against their potential cardiopulmonary risks. By doing so, the study aims to inform clinical decision-making and support the delivery of safer, more effective care in managing ALK-rearranged malignancies.

## Methods

The methods have previously been described [14,15]. The FAERS database is a publicly accessible, spontaneous reporting system maintained by the U.S. FDA that collects adverse event (AE) reports related to drugs and therapeutic biologics from multiple sources, including healthcare providers, pharmaceutical manufacturers, and consumers both domestically and internationally. It comprises several datasets covering patient demographics, drug information, adverse event coding, outcomes, report sources, therapy dates, and indications, all coded according to standardized medical terminologies such as MedDRA.The database is continually updated, enabling ongoing post-marketing safety surveillance and early detection of potential drug safety signals.

For this study, data from January 1, 2013, to December 31, 2023, were extracted and analyzed using OpenVigil 2.1, a web-based pharmacovigilance tool that facilitates querying FAERS and applies rigorous data cleaning protocols, including deduplication of reports, correction of formatting errors, and standardization of drug names to ensure data quality and consistency. Only reports where the drug was designated as the “primary suspect” were included to strengthen the attribution of adverse events to the drug of interest.

Disproportionality analysis was employed to identify signals of disproportionate reporting of specific adverse events associated with crizotinib, ceritinib, alectinib, brigatinib, and lorlatinib compared to the background reporting frequency of these events with all other drugs, including other kinase inhibitors in the FAERS database. The primary statistical measure used was the Reporting Odds Ratio (ROR), calculated from a 2 × 2 contingency table that cross-classifies reports by presence or absence of the AE and exposure or non-exposure to the drug. An ROR greater than 1 indicates a higher-than-expected reporting frequency, suggestive of a potential association, though not proof of causality. Statistical significance was defined as an ROR exceeding 2.00 with the lower bound of the 95% confidence interval above 1.00, and only AEs with at least ten reports were considered to ensure robustness. This threshold aligns with established pharmacovigilance criteria to minimize false positives while identifying meaningful safety signals.

Adverse events were classified according to MedDRA preferred terms and system organ classes, facilitating standardized categorization and interpretation. The analysis focused on spontaneous reports where the kinase inhibitors were the primary suspect drugs, enhancing specificity of the findings. Ethics approval and patient consent were not required as the study utilized publicly available data collected by the U.S. FDA.

## Results

The ROR and the number of reports for pleural and pericardial effusions associated with ALK inhibitors included in the study are summarized in Table 1. For crizotinib, pleural effusion was reported with an ROR of 7.76 (95% CI: 6.60–9.12) across 150 reports, while pericardial effusion had a higher ROR of 9.00 (95% CI: 7.10–11.41) with 69 reports. Ceritinib exhibited similar trends, with pleural effusion having a ROR of 7.36 (95% CI: 5.16–10.50) based on 31 reports. Pericardial effusion had a ROR of 10.80 (95% CI: 6.79–17.19) from 18 reports. For alectinib, pleural effusion had an ROR of 4.76 (95% CI: 3.80–5.97) from 76 reports, while pericardial effusion had an ROR of 6.67 (95% CI: 4.92–9.04) from on 42 reports. Brigatinib had higher RORs for both pleural effusion at 8.70 (95% CI: 6.58–11.52) and pericardial effusion at 7.87 (95% CI: 4.95–12.51) from 50 and 18 reports respectively. Lastly, lorlatinib presented the highest RORs among the drugs in the study, with pleural effusion having an ROR of 8.61 (95% CI: 6.72–11.02) from 64 reports, and pericardial effusion having an ROR of 12.57 (95% CI: 9.08–17.38) from 37 reports. These findings suggest variable but notable associations between ALK inhibitors and the occurrence of effusions, with lorlatinib showing the strongest associations in both pleural and pericardial.

**Table 1:** Statistical data showing the association between ALK inhibitors and pleural/pericardial effusions.

Drug	Reporting Odds Ratio (95% Confidence Interval)	Number of Reports
Crizotinib		
Pleural Effusion	7.76 (6.60, 9.12)	150
Pericardial Effusion	9.00 (7.10, 11.41)	69
Ceritinib		
Pleural Effusion	7.36 (5.16, 10.50)	31
Pericardial Effusion	10.80 (6.79, 17.19)	18
Alectinib		
Pleural Effusion	4.76 (3.80, 5.97)	76
Pericardial Effusion	6.67 (4.92, 9.04)	42
Brigatinib		
Pleural Effusion	8.70 (6.58, 11.52)	50
Pericardial Effusion	7.87 (4.95, 12.51)	18
Lorlatinib		
Pleural Effusion	8.61 (6.72, 11.02)	64
Pericardial Effusion	12.57 (9.08, 17.38)	37

## Discussion

The findings of this study emphasize a significant association between ALK inhibitors and the development of pleural and pericardial effusions, with notable variability among individual drugs. Lorlatinib exhibited the strongest association with both AEs. Crizotinib, brigatinib, and ceritinib demonstrated similarly high associations. At the same time, alectinib had comparatively lower RORs for both types of effusions, though still statistically significant. These results underscore the need for heightened clinical vigilance and proactive management of effusion-related complications in patients undergoing treatment with these agents. By understanding the differential risk profiles of each ALK inhibitor, clinicians can make informed decisions tailored to individual patient needs, balancing the therapeutic benefits against potential cardiopulmonary risks.

Crizotinib, the first ALK inhibitor approved for clinical use, significantly altered the treatment paradigm for ALK-rearranged malignancies [16,17]. In this study, it was demonstrated to be associated with both pleural and pericardial effusions, confirming prior observations of cardiopulmonary toxicity [18,19]. Of note, crizotinib also inhibits other kinases, including MET and ROS1, which may contribute to its toxicity profile which includes heart failure, arrhythmias, and conduction abnormalities [20–23]. Ceritinib, a second-generation ALK inhibitor, has provided a second-line option for patients with crizotinib resistance [24]. However, it also showed a notable association with pleural effusion and an even stronger association with pericardial effusion. The broader kinase polypharmacy effect of ceritinib may account for its higher toxicity in this regard as well [25]. Despite these risks, ceritinibs potency in overcoming ALK resistance mutations underscores its value, especially in advanced or refractory cases. Brigatinib, another second-generation ALK inhibitor, demonstrated significant RORs for both effusions. Brigatinib is particularly effective against ALK mutations conferring resistance to earlier-generation inhibitors, making it indispensable in treating advanced disease [26]. However, its similar toxicity profile necessitates a nuanced approach to patient management. Alectinib demonstrated the lowest RORs for both effusions among the drugs included in the study. Despite its comparatively lower risk, the occurrence of effusions still surpasses the background rates observed with other therapies, indicating the need for ongoing surveillance. Alectinib’s safety and efficacy profile make it a suitable choice for frontline therapy, and its tolerability may enhance long-term adherence in patients with ALK-rearranged malignancies. Lorlatinib exhibited the highest RORs for both effusions, identifying it as the ALK inhibitor most strongly associated with these adverse events. Lorlatinib’s broad-spectrum activity against a wide range of ALK resistance mutations is a critical factor in its clinical utility [27]. The mechanisms driving its strong association with effusions may involve widespread kinase inhibition as previously demonstrated [28]. Given these risks, lorlatinib should be used with caution, particularly in patients with pre-existing cardiopulmonary conditions. Proactive monitoring through imaging studies and laboratory assessments is essential for patients displaying relevant symptoms, and early interventions should be implemented at the first signs of effusion-related symptoms. Despite these challenges, lorlatinib’s unparalleled efficacy in treating resistant ALK-positive malignancies ensures its continued relevance in clinical practice, even for patients with cardiopulmonary risk.

These complications arise from a combination of pharmacological mechanisms and off-target effects, highlighting the importance of pharmacovigilance in monitoring and managing these events. Disproportionality analyses using global pharmacovigilance databases, such as VigiBase and FAERS, have provided evidence linking ALK inhibitors to cardiovascular toxicities, including pericarditis and associated pericardial effusions. For example, all five approved ALK inhibitors exhibited significantly elevated RORs for pericarditis in VigiBase [29]. Crizotinib and lorlatinib, in particular, have been implicated in heart failure, which can contribute to secondary pericardial effusions due to reduced cardiac output and increased central venous pressure [19]. Moreover, these effects can manifest early in the treatment course, with the median onset of cardiac-related adverse events reported within two months of therapy initiation. This underscores the importance of early and ongoing monitoring of cardiac function in patients receiving ALK inhibitors.

Pharmacovigilance studies have also highlighted the need to consider drug-specific profiles when evaluating the risk of effusions. For example, crizotinib, the first-generation ALK inhibitor, has been linked to a broader range of cardiovascular toxicities, while newer agents like alectinib and brigatinib may exhibit varying degrees of cardiotoxicity. Understanding these differences is critical for tailoring risk mitigation strategies, such as dose modifications, closer monitoring of fluid retention, and early intervention when signs of effusions emerge. The multifactorial nature of effusions in patients treated with ALK inhibitors necessitates a nuanced approach to pharmacovigilance. Comprehensive adverse event reporting and analysis enable the identification of patterns, such as dose-dependent toxicity, early onset, or exacerbation by comorbid conditions. Integrating pharmacological data with clinical outcomes can inform guidelines for monitoring and managing these adverse events, ensuring that the therapeutic benefits of ALK inhibitors are not undermined by preventable complications. Further research into the molecular pathways driving endothelial dysfunction and cardiotoxicity is essential to optimize the safety profile of these life-extending agents.

The FAERS database is a valuable resource for identifying potential AE patterns related to ALK inhibitors but has limitations that need discussion. Underreporting is a significant concern, as many adverse events may not be captured. Reporting biases, such as the preferential submission of severe cases, can distort the data and reduce its generalizability. Additionally, because FAERS data is observational, it cannot establish causality, only associations, necessitating cautious interpretation of potential links between ALK inhibitors and pleural or pericardial effusions. The reliance on RORs presents further challenges, as RORs indicate possible drug-AE relationships without proving causality or accounting for confounding factors. Differentiating AEs specific to ALK inhibitors from those of other treatments is complicated by overlapping mechanisms of drug toxicity. These limitations highlight the need for further research and robust datasets to confirm associations and elucidate underlying mechanisms. Despite these constraints, the findings of this analysis offer a critical foundation for enhancing patient care. Integrating these insights into clinical practice enables healthcare providers to develop targeted strategies for managing the risks associated with ALK inhibitors. Identifying high-risk AEs, such as pleural and pericardial effusions, supports evidence-based protocols for monitoring and intervention. Proactive approaches, including routine imaging for early detection of effusions, vigilant monitoring of respiratory and cardiac status, and timely therapeutic management, can significantly improve patient outcomes. Furthermore, a multidisciplinary care model involving oncologists, cardiologists, and pulmonologists is crucial to provide comprehensive care, balancing the therapeutic benefits of ALK inhibitors with the management of potential adverse effects.

This study highlights the significant association between ALK inhibitors and the development of pleural and pericardial effusions, with notable variability across individual agents. Lorlatinib exhibited the strongest associations, followed by crizotinib, brigatinib, and ceritinib, while alectinib demonstrated the lowest RORs. These findings underscore the importance of vigilant monitoring and proactive management of effusion-related adverse events in patients receiving ALK inhibitors. Despite their potential risks, ALK inhibitors remain key in the treatment of ALK-rearranged malignancies, providing substantial oncologic benefits. A balanced approach to therapy, informed by the differential risk profiles of each agent, can optimize patient outcomes while minimizing adverse effects. Further research is needed to elucidate the mechanisms underlying these associations and to develop targeted strategies for mitigating toxicity, ultimately supporting the safe and effective use of ALK inhibitors in clinical practice.
